# A Longitudinal Study of Interprofessional Education Readiness and Collaboration Skills Among Japanese Psychology Undergraduates

**DOI:** 10.7759/cureus.86021

**Published:** 2025-06-14

**Authors:** Ayako Hase, Junko Kawahito, Norifumi Tsuno, Masanao Yokohira, Nobuyasu Komasawa, Ikuko Kato, Shuji Noguchi, Tomomi Shinohara, Kaoru Tsuge, Miyuki Uematsu, Motohiko Takemori, Kenji Kanbara

**Affiliations:** 1 Department of Clinical Psychology, Faculty of Medicine, Kagawa University, Miki-cho, JPN; 2 Department of Medical Education, Faculty of Medicine, Kagawa University, Miki-cho, JPN; 3 Community Medicine Education Promotion Office, Faculty of Medicine, Kagawa University, Miki-cho, JPN; 4 Department of Nursing, Faculty of Medicine, Kagawa University, Miki-cho, JPN; 5 Department of Neuropsychiatry, Kagawa University Hospital, Miki-cho, JPN; 6 Department of Medical Oncology, Kagawa University Hospital, Miki-cho, JPN; 7 Department of Psychiatry and Psychosomatic Medicine, Sanuki City Hospital, Sanuki-shi, JPN

**Keywords:** interprofessional collaboration skills, interprofessional education (ipe), licensed psychologists, longitudinal study, readiness

## Abstract

Introduction: Few longitudinal studies have examined the effects of interprofessional education (IPE), particularly those involving psychology students. In Japan, where national certification for psychologists was established in 2017, a common challenge faced by psychologists is the content and methods of IPE.

Objective: A four-year longitudinal study was conducted to examine the sustained effects of first-year IPE within an undergraduate training program for licensed psychologists. In addition, a separate study investigated the changes in interprofessional work skills in a non-IPE practicum.

Methods: This study employed a mixed-method research design combining (i) a longitudinal observational study (2020-2023) to examine the sustained effects of the IPE implemented in the 2020 academic year and (ii) a quasi-experimental pre-post test study independently conducted with the same participants in the 2023 academic year. The design was based on (i) Kolb's Experiential Learning Model and (ii) Schön's Reflective Practitioner Model, and one of the goals was to develop interprofessional work skills. The participants were students in a licensed psychologist training program who enrolled in 2020. We investigated their readiness for IPE (using the Readiness for Interprofessional Learning Scale (RIPLS)) over a four-year period (primary evaluation) and interprofessional competency (using the Interprofessional Competency Assessment Scale for Undergraduates (ICASU)) before and after their fourth year of practice (secondary evaluation).

Results: RIPLS scores from first to fourth year neither improved nor declined; ICASU scores increased from 56.00 (49.00-63.00) before the program to 63.00 (55.00-67.00) after the program (p = 0.005). On the subscales, “understanding one’s own and other occupations” (p = 0.013) and “interprofessional collaboration skills” (p = 0.014) improved significantly. Regarding “basic communication skills,” which did not improve significantly, the results of the item-by-item analysis showed that items related to recognizing diverse values (p = 0.008) and finding one's issues (p = 0.003) improved; however, four items, including assertive discussion skills, did not improve.

Conclusion: In the longitudinal survey, readiness for IPE did not decrease after the first year of IPE. This suggests that even without continuous IPE, readiness for IPE could be maintained, and the basic skills for interprofessional work could be developed by devising a curriculum and practice program.

## Introduction

Collaborative practice among healthcare professionals is essential for delivering high-quality care in increasingly complex healthcare settings, where various professionals work toward common goals while contributing their specialized knowledge [1].

Interprofessional education (IPE) is defined as an educational approach wherein individuals from two or more professional backgrounds learn with, from, and about each other to develop effective collaboration skills and improve health outcomes [1]. In professional training programs, IPE plays a central role in cultivating the fundamental competencies required for effective interprofessional teamwork.

According to a survey on IPE in Japan, approximately 90% of medical schools require IPE programs [2]. Of these, approximately half were offered during the first two years of study. In many studies, the readiness for IPE has been improving [3,4]. However, few universities have systematically and progressively promoted IPE in the early grades, and even fewer longitudinal studies have reported lasting effects after IPE implementation.

Globally, IPE is open to students from a wide range of disciplines, including medicine, nursing, pharmacy, dentistry, physical therapy, and occupational therapy [1]. However, there are only a few reports of IPE involving psychology majors [5]. Previous studies on IPE, including psychology majors, have shown that the readiness for IPE is lower than that of students in other majors [6-8].

In Japan, the first national certification system for psychologists was established in 2017. The new nationally licensed psychologist is expected to use broad psychological knowledge and skills, including those from basic psychology, health psychology, and clinical psychology, to provide psychological support. The role of licensed psychologists includes not only providing psychological assessment and psychotherapy but also contributing to the team, including supporting family members and colleagues while collaborating with other professionals.

Licensed psychologists are required to complete a four-year undergraduate training course and a two-year master's training course, which does not constitute a continuous six-year program. As such, it is not always possible to obtain consistent education at a single institution [9]. Moreover, licensed psychologists are not necessarily employed in the medical field; they also work in various sectors such as education, welfare, and the judicial system. While some studies [10] have examined first-year IPE in Japanese licensed psychologist training programs, longitudinal evaluations of its long-term effects are lacking.

In the training program at Kagawa University, to which the authors belong, IPE is conducted during the first year but not thereafter. This raises important questions: Can IPE readiness be maintained in the absence of ongoing IPE? Is it possible to foster interprofessional competence even within practicum settings that do not explicitly include IPE?

A systematic review [11] of the effectiveness of IPE revealed positive outcomes regarding IPE readiness and interprofessional competency in approximately half of the included articles. However, 65% of the papers included were conducted in the first half of the curriculum, and approximately 70% were cross-sectional studies. In addition, since the review included many single activities, longitudinal studies are needed to examine long-term interventions and the persistence of their effects.

The primary aim of this study was to evaluate the sustained effects of IPE implemented during the first year of the psychology program at Kagawa University, to which the authors are affiliated, on students’ readiness for IPE. The secondary objective was to examine changes in interprofessional collaboration skills before and after the fourth-year clinical training (which did not include IPE) in the same group of students. These two studies were conducted independently.

Given that this research focuses on a clinical training program conducted in medical settings, it is primarily situated within the framework of clinical psychology. This perspective reflects the practical competencies expected of nationally licensed psychologists in Japan, who are required to integrate broad psychological knowledge, including health, behavioral, and clinical psychology, into multidisciplinary healthcare contexts.

## Materials and methods

Curriculum

The licensed psychologist training program at Kagawa University, which was the focus of this study, is conducted within the Faculty of Medicine - an arrangement that is rare in Japan. The undergraduate program admits 20 students per year, all of whom complete the training course for certified psychologists. Most students pursue master's degree programs at universities or other graduate schools in Japan to become licensed psychologists. The university places particular emphasis on fostering readiness for interprofessional collaboration, which is also reflected in its educational philosophy. Additionally, the university offers a comprehensive range of lectures and practical training courses delivered by specialists in other professions. Table 1 shows the statutory subjects and number of hours stipulated by the national government.

**Table 1:** Courses that represent the uniqueness of the university compared to statutory courses

Courses Required by Law	Courses at Kagawa University
Structure and Functions of the Human Body and Diseases (one course)	Structure and Functions of the Human Body and Diseases (five courses): Introduction to Medicine, Introduction to Medical Physiology, Introduction to Anatomy, Introduction to Biochemistry and Molecular Biology, Outline of Medical Disease for Clinical Psychologists
Psychological Practice (80 hours or more)	Psychological Practice (150 hours): Psychological Practice I, Psychological Practice Ⅱ (Team Medicine Care)
Undefined	Unique courses: Early Exposure to Medicine (IPE), Growth and Development of Childhood, Disaster Medicine, Palliative and Supportive Care, Psychosomatic Medicine, Bioethics, Clinical Pharmacology, Introduction to Pathology and Immunology, Introduction to Microbiology and Medical Zoology

Practicum program

The training program for licensed psychologists at Kagawa University incorporates IPE during the first year of the undergraduate program but not thereafter. However, the program aims to foster interprofessional competency through a curriculum and practical training that capitalizes on its unique features. In particular, “Team Medicine Care," a practical training course conducted in the fourth year, is a subject that embodies this philosophy. The details are given below.

“Early Exposure to Medicine" course during the first year is an IPE with the Faculty of Medicine. The content includes lectures, exercises (group work), and practical training (Table 2). In 2020, the year that was the subject of this study, off-campus training was reduced due to COVID-19, and the original four-day schedule was shortened to one to two days.

**Table 2:** “Early Exposure to Medicine” implementation plan for 2020

Steps	Contents
1-3	Orientation and basic lectures on the body and facilities
4-6	Explanation of practical training and survey of desired practical training facilities
7-8	Group work on practical training
9-12	Practice I (two days; shortened due to COVID-19)
15-18	Practice II (two days; shortened due to COVID-19)
19-27	Preparation for presentation of results
29-30	Presentation of practice results

“Team Medicine Care” course in the fourth year is a non-IPE practical course, as it is exclusively open to students majoring in psychology. It comprises a field trip to a hospital affiliated with the Faculty of Medicine, supportive practice at a community hospital (involving direct contact with patients), and on-campus instruction (Table 3), conducted in small groups of three to four students on a rotating basis. The practicum program was designed by researchers based on (i) the Experiential Learning Model [12] and (ii) the Reflective Practitioner Model [13], with the aim of enhancing interprofessional competency.

**Table 3:** “Team Medicine Care” implementation plan for 2023

Steps	Contents
1-3	Orientation
4-7	Team Medicine lecture by the multidisciplinary staff of the affiliated hospital
8-11	Psychiatric Neurology practice
12	Group reflection
13-20	Community Hospital practice
21	Group reflection
22-25	Pediatric practice
26	Group reflection
27-28	Medical Oncology practice
29	Group reflection
30	Presentation of practice results

**Figure 1:**
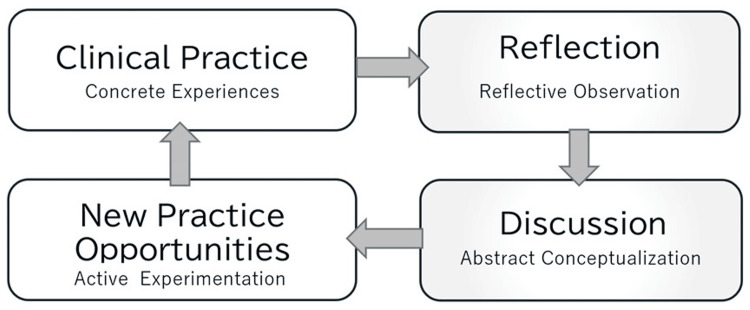
Circular structure of the "Team Medicine Care” program This figure was developed by the authors based on Kolb’s Experiential Learning Cycle [12].

First, the structure of the “Team Medicine Care” program was designed in alignment with Kolb’s Experiential Learning Cycle, which consists of four stages: concrete experience, reflective observation, abstract conceptualization, and active experimentation [12]. Specifically, clinical practices such as psychiatric neurology or community hospital rotations serve as concrete experiences, followed by reflective observation sessions. These reflections lead to abstract conceptualization, which is supported by lectures and group discussions, and further reinforced through active experimentation in subsequent clinical settings. Figure 1 illustrates this cyclical process.

Next, based on Schön's Reflective Practitioner Model [13], the content of the reflections was structured. This model describes a growth model in which practising professionals in the field, when encountering unexpected situations, “dialogue with the situation,” engage in “reflection in action,” and derive a theory of practice. During this process, verbalizing the situation and discussing it with others is essential. In the “Team Medicine Care” course, on-campus reflection sessions are conducted in joint groups of about 10 students, where each individual shares a “memorable episode” under the guidance of a faculty member, followed by a collective discussion of the episode.

Subsequently, a program was developed to enhance interprofessional competency - an essential component of IPE, which is defined as "occasions when two or more professions learn with, from, and about each other to improve collaboration and the quality of care” [1]. In pre-graduate education, “two or more professions” typically refers to students from different academic departments, but IPE is not inherently limited to training programs or students. The interaction and participation of professionals with students at the training site have a bidirectional educational effect in terms of deepening their understanding of their own professional collaboration [14]. In the “Team Medicine Care” program, to foster interprofessional competency, a “Team Medicine" lecture by multidisciplinary staff from the affiliated hospital is held at the beginning of the practicum. The lectures are delivered by professionals from 12 different fields (Table 4) and include interactive components such as question-and-answer (Q&A) sessions, discussions with group members, submission of reports, and feedback from the lecturers. Additionally, daily Q&A sessions with lecturers and other trainees are encouraged during field training.

**Table 4:** Professionals in 12 different fields

Professional fields
Midwife, Pediatric Emergency Nurse, Disaster Nurse, Palliative Care Nurse, Liaison Nurse, Physical Therapist, Occupational Therapist, Speech-Language Pathologist, Dietitian, Pharmacist, Medical Social Worker, Health Insurance Claims Specialist

Study participants

The present longitudinal study initially included 21 students who were enrolled as of April 2020. As the study was designed to follow a predefined cohort over four years, no a priori sample size calculation was applicable. Of these, students who were absent on the day of data collection, had taken a leave of absence or withdrawn from the program, or whose data were incomplete, were excluded from the analysis. Consequently, the final analytical sample comprised 15 students with complete data across the four-year observation period (Table 5). It should be noted that the same 15 students who comprised the final analytical sample in the longitudinal study were also included in the analysis of the effects before and after the fourth-year clinical practicum.

**Table 5:** Demographic information of the participants Four-Year Continuous Valid Respondents (%) refers to the number (and percentage) of participants who consistently responded over all four years based on the initial sample size. IPE: Interprofessional education; RIPLS: Readiness for Interprofessional Learning Scale; ICASU: Interprofessional Competency Assessment Scale for Undergraduates

Scale		Undergraduate Year 1 (Academic Year 2020) - Pre IPE	Undergraduate Year 1 (Academic Year 2020) - Post IPE	Undergraduate Year 2 (Academic Year 2021)	Undergraduate Year 3 (Academic Year 2022)	Undergraduate Year 4 (Academic Year 2023) - Pre Non-IPE	Undergraduate Year 4 (Academic Year 2023) - Post Non-IPE	Four-Year Continuous Valid Respondents (%)
RIPLS	Subjects	21	21	21	21	-	21	15 (71.4)
	Distributed survey sheets (excluding absentees, leaves of absence, and withdrawals)	21	19	19	17	-	19	
	Valid responses	21	19	19	17	-	19	
ICASU	Subjects	-	-	-	-	21	21	-
	Distributed survey sheets (excluding absentees, leaves of absence, and withdrawals)	-	-	-	-	19	19	
	Valid responses	-	-	-	-	19	19	

Study design and setting

This study utilized a mixed-method research design, combining (i) a longitudinal observational study (2020-2023) to examine the sustained effects of the IPE implemented in the 2020 academic year and (ii) a quasi-experimental pre-post test study independently conducted with the same participants in the 2023 academic year. All components followed a quantitative approach. 

The longitudinal study proceeded as follows: During the first year of undergraduate studies (academic year 2020), surveys were administered both at the beginning and the end of the IPE program. In the second (2021) and third (2022) years, surveys were conducted at the end of each academic year. In the fourth year (2023), surveys were administered after practicum training. 

The same questionnaire was used for the surveys conducted before and after IPE in the first year (2020), as well as at the end of the second (2021) and third (2022) years. This questionnaire was constructed based on RIPLS and did not incorporate ICASU. In the fourth year (2023), the surveys conducted before and after the practicum training included both RIPLS and ICASU. 

The study evaluating the educational impact of the fourth-year (2023) practicum training using RIPLS and ICASU was conducted as a separate research project, independent from the longitudinal study employing RIPLS. 

Variables

The primary outcome measure was RIPLS, a tool widely used worldwide to evaluate international IPE. The Japanese version [15] has been translated, and its reliability and validity have been confirmed. It is publicly available, and its use is permitted. RIPLS is a 19-item scale that measures orientation toward IPE and consists of three subcategories. The categories are as follows: (i) "teamwork and collaboration" (13 items; range 13-65), (ii) "IPE opportunities" (two items; range 2-10), and (iii) "uniqueness of profession" (four items; range 4-20). Each item is answered on a five-point Likert scale, with responses ranging from “strongly disagree" (1) to "strongly agree" (5).

ICASU [16], developed in Japan, is a publicly available assessment tool with confirmed reliability and validity, and its use is permitted. In the present study, it was employed as a secondary outcome measure. ICASU is based on the three core competencies proposed by Barr [17]: "basic communication skills” (six items; range 6-30), “understanding one's own and other occupations” (four items; range 4-20), and "interprofessional collaboration skills” (six items; range 6-30), which have been improved by IPE in previous studies [16,18]. Each item is answered on a five-point Likert scale, with responses ranging from “strongly disagree" (1) to "strongly agree" (5).

Statistical analysis

Statistical analyses were performed using IBM SPSS Statistics for Windows, Version 28.0 (IBM Corp., Armonk, USA). The Cronbach's alpha coefficient was calculated to assess the internal consistency of the RIPLS and ICASU. The comparison of average RIPLS scores of students over four years was analyzed using the Friedman test. The comparison of ICASU scores before and after the practical training was made using the Wilcoxon signed-rank test. Continuous data were presented as medians and 25%-75% tiled values. Given the small sample size and the potential for non-normal data distribution, we used non-parametric tests (Friedman and Wilcoxon signed-rank tests) for statistical analysis. Consequently, data were presented as median and interquartile range (IQR). Statistical significance was set at p < 0.05.

Ethical considerations

This study was conducted with the approval of the Ethics Committee of the Faculty of Medicine, Kagawa University. Initially, ethical approval was obtained for a four-year longitudinal study examining temporal effects (No. 2020-0149), followed by approval for a study investigating the outcomes of the non-IPE practicum in the fourth year (No. 2023-010). Subsequently, approval was granted for the present study, which integrates the research components (No. 2024-150).

Informed consent was obtained verbally by the principal investigator in the presence of other faculty members. Additionally, a consent statement was included at the beginning of the questionnaire. All participants were provided with a thorough explanation of the study’s objectives and procedures. We emphasized that not participating in the study would not affect grades or advancement. Although student identification numbers were collected to facilitate longitudinal analysis, data were anonymized and managed using alternative ID codes to ensure confidentiality. It was explicitly communicated that participation in the study and questionnaire responses would not impact academic performance. Furthermore, although the study employed a longitudinal and pre-post design, participants were informed of their right to withdraw at any point without any disadvantage.

## Results

Longitudinal changes in RIPLS

Cronbach's alpha for all RIPLS items was 0.90, with “teamwork and collaboration” at 0.91, “IPE opportunities” at 0.86, and “uniqueness of profession” at 0.47. Table 6 presents the RIPLS scores collected over the four-year longitudinal study (2020-2023). The Friedman test revealed no statistically significant changes in the scores across the four time points.

**Table 6:** Median RIPLS in each year and p-value from the Friedman test (N = 15) Data are presented as median (IQR). A p-value < 0.05 was considered statistically significant. IQR: Interquartile range; IPE: Interprofessional education; RIPLS: Readiness for Interprofessional Learning Scale

Subscale	Undergraduate Year 1 (Academic Year 2020) - Pre IPE	Undergraduate Year 1 (Academic Year 2020) - Post IPE	Undergraduate Year 2 (Academic Year 2021)	Undergraduate Year 3 (Academic Year 2022)	Undergraduate Year 4 (Academic Year 2023)	X^2^-value	P-value
	Median (IQR)	Median (IQR)	Median (IQR)	Median (IQR)	Median (IQR)		
Total	86.00 (81.00-88.00)	87.00 (81.00-89.00)	87.00 (76.00-89.00)	85.00 (76.00-91.00)	85.00 (80.00-91.00)	1.47	0.833
Teamwork and collaboration	61.00 (57.00-64.00)	64.00 (57.00-64.00)	62.00 (56.00-65.00)	61.00 (55.00-65.00)	60.00 (55.00-64.00)	1.63	0.804
IPE opportunities	10.00 (9.00-10.00)	10.00 (9.00-10.00)	10.00 (8.00-10.00)	10.00 (9.00-10.00)	10.00 (10.00-10.00)	7.73	0.102
Uniqueness of profession	15.00 (14.00-17.00)	14.00 (13.00-16.00)	15.00 (14.00-16.00)	14.00 (12.00-18.00)	15.00 (14.00-17.00)	4.95	0.292

Changes in ICASU before and after practice

Cronbach's alpha coefficients for all ICASU subscales were as follows: overall, 0.93; “basic communication skills,” 0.77; “understanding one's own and other occupations,” 0.84; and “interprofessional collaboration skills,” 0.92. Table 7 displays the ICASU scores measured before and after the 2023 practicum intervention. The Wilcoxon signed-rank test indicated a statistically significant improvement in the total score following the intervention. On the subscales, “understanding one's own and other occupations” and “interprofessional collaboration skills” showed significant improvements, whereas “basic communication skills” did not show a statistically significant change.

**Table 7:** Median ICASU and Wilcoxon signed-rank test results (N = 15) Data are presented as median (IQR). A p-value < 0.05 was considered statistically significant. (*) indicates statistical significance. IQR: Interquartile range; IPE: Interprofessional education; ICASU: Interprofessional Competency Assessment Scale for Undergraduates

Subscale	Undergraduate Year 4 (Academic Year 2023) - Pre Non-IPE	Undergraduate Year 4 (Academic Year 2023) - Post Non-IPE	Z-value	P-value
	Median (IQR)	Median (IQR)		
Total	56.00 (49.00-63.00)	63.00 (55.00-67.00)	-2.28	0.005^*^
Basic communication skills	24.00 (22.00-28.00)	25.00 (23.00-27.00)	-1.31	0.190
Understanding one’s own and other occupations	13.00 (12.00-16.00)	15.00 (13.00-17.00)	-2.48	0.013^*^
Interprofessional collaboration skills	19.00 (15.00-22.00)	21.00 (19.00-25.00)	-2.45	0.014^*^

To explore this further, Wilcoxon signed-rank tests were conducted for each item of “basic communication skills” (Table 8). The results showed that two items related to the recognition of diverse values and the identification of one's own issues improved, but four items related to assertive discussion skills did not.

**Table 8:** Medians and Wilcoxon signed-rank test results for each item of the ICASU subscale of “basic communication skills” (N = 15) Data are presented as median (IQR). A p-value < 0.05 was considered statistically significant. (*) indicates statistical significance. IQR: Interquartile range; IPE: Interprofessional education; ICASU: Interprofessional Competency Assessment Scale for Undergraduates

Question Item	Pre Non-IPE	Post Non-IPE	P-value
	Median (IQR)	Median (IQR)	
(1）Can hold a conversation with consideration of the time and circumstances.	4.00 (4.00-5.00)	4.00 (4.00-5.00)	0.739
(2）Can make observations with due regard to time and place.	4.00 (4.00-5.00)	5.00 (4.00-5.00)	0.059
(3）Be aware of diverse values and ways of thinking through interacting with others.	4.00 (4.00-5.00)	5.00 (4.00-5.00)	0.008^*^
(4）Can express one's own opinion about differences in thinking based on the other person's position.	4.00 (3.00-5.00)	4.00 (3.00-5.00)	0.782
(5）Can discuss differences of opinion and find valid points.	4.00 (3.00-4.00)	4.00 (3.00-4.00)	0.782
(6）Be aware of one's own tendencies, issues, and characteristics in membership through group work.	4.00 (3.00-5.00)	5.00 (4.00-5.00)	0.003^*^

## Discussion

Many intervention studies on pre- and post-IPE using the RIPLS have reported improved scores [11]. Some longitudinal studies examining the educational effects of IPE have reported that RIPLS scores decrease with increasing grades [19,20].

The results of this study showed no significant decline in readiness for IPE over the four years. Previous longitudinal survey studies considered factors such as low expectations of IPE at the time of admission, poor interaction with students of other majors outside IPE [19], and reinforced negative values toward learning from other professionals [20] as background factors for the decline or stagnation in readiness for IPE. Since the educational philosophy of the university is to nurture individuals who can practice interprofessional collaboration, the expectations for IPE of the incoming students are high. In addition, the campuses and curricula of the university provide students with opportunities to interact with staff of other professions and students of other majors through lectures and extracurricular activities. These background factors may have contributed to the fact that the RIPLS scores did not decrease significantly.

In this study, changes in interprofessional collaboration skills during non-IPE practicum in the fourth year were investigated as a secondary evaluation, and significant improvements were found in the total score, “understanding one's own and other occupations,” and "interprofessional collaboration skills.”

In “understanding one's own and other occupations,” professional identity is developed in the academic year in which the professional curriculum is advanced, through the experience of field observations and discussions with other professions [16]. The “Team Medicine Care” course in this study is also a practice in the fourth year, when the professional curriculum has advanced. Although it cannot be said to be an experience equivalent to IPE, it includes a program that actively incorporates dialogue with staff from other professions. Practical training has an interactive and multidirectional educational effect in that staff members reflect on their own attitudes toward professional collaboration and deepen their understanding through dialogue with students. Therefore, it is possible that the program design, taking into consideration the grade level of the program and emphasizing dialogue with staff of other professions, contributed to the improvement in the "understanding one's own and other occupations” score.

“Interprofessional collaboration skills” assess the ability to collaborate with practical teams. It has been considered that this ability may be improved through collaborative activities with patients and other professionals, especially in the final year of the program [16]. The “Team Medical Care” program at the university is not just a simple observation internship, but includes interaction with patients and other staff members. After observing and interacting with them, students participate in a Q&A session with a supervisor about collaborative practice in their profession, and then discuss the topic in small groups. This verbalization of concepts through discussion enhances the effectiveness of observational learning [21]. In fact, past studies have reported that verbalizing experiences through discussion after observational learning improves learning outcomes [22,23]. In particular, in the field of medical education, observing collaborative practices by instructors and then engaging in verbalization and reflection has been shown to deepen understanding of team-based medicine and foster collaborative attitudes [24]. Based on these findings, the internship training in this study may have contributed to the improvement of "interprofessional collaboration skills."

In terms of “basic communication skills,” the results of the item-by-item analysis showed significant improvements in "being able to recognize diverse values and ways of thinking when interacting with others" (p = 0.008) and "being able to recognize one's tendencies, issues, and characteristics in membership through group work" (p = 0.003). Although reflection is emphasized in IPE, its method has not yet been established [25]. In this study, it is possible that the participants became aware of diverse values and contributed to discovering their awareness and issues by discussing episodes with members that had a strong emotional impact following the reflective practitioner model.

No significant changes were observed in the remaining four items, such as “can hold a conversation with consideration of the time and circumstances” and “can express one's own opinion about differences in thinking based on the other person's position.” Critical thinking skills need to be nurtured to facilitate reflective practice in interprofessional collaboration [25]. It is necessary to find valid points in each other's opinions through reflection and to seek discussion methods that foster an assertive attitude. The same results were shown in a previous IPE study [18] regarding the changes in “conversing and observing with an awareness of the situation” through practical training, and we believe it is necessary to examine these items in the future.

Limitations and future directions

The results of this study showed that readiness for IPE did not decrease after the first year of IPE implementation and that interprofessional collaboration skills improved in the fourth year of non-IPE practicum. This study holds significant global value as it longitudinally examines the development of interprofessional collaboration competencies among students majoring in psychology. Notably, the implementation of IPE targeting psychology students is exceptionally challenging in Japan and is seldom practiced. The findings of this research demonstrate the possibility of cultivating interprofessional collaboration skills even through practicum experiences classified as non-IPE, which may have a considerable impact on the curriculum models adopted by other universities.

However, this study has several limitations. Since previous studies on longitudinal surveys did not describe the content of IPE in detail, we could not conduct a comparative study with the curriculum and practice at our university. In addition, since we did not conduct comparative studies with universities that do not offer first-year IPE, we cannot discuss the relationship between the persistence effect of IPE and improvements in interprofessional collaboration skills. Many previous studies have pointed out that introducing IPE early in a healthcare course may help break through negative attitudes and avoid stereotyping [2]. Future qualitative studies should discuss the lasting effects of first-year IPE.

Otherwise, owing to the small sample size and bias, it is highly possible that the background factors of the target student population influenced the results as confounding factors. The validity and reliability issues of RIPLS have been pointed out [26,27], and in the Japanese version of RIPLS, consistency and reliability issues have been pointed out, especially in the “uniqueness of profession” subscale [15]. As the same tendency was observed in this study, it is necessary to accumulate RIPLS data in Japan and simultaneously examine the content and structure of the items. The RIPLS and ICASU are based on self-assessment, which may be influenced by social desirability bias. A previous domestic study [18] of IPE, after acknowledging the limitations of the self-assessment finger, used it based on its usefulness in assessing problem-solving skills and promoting deep and long-lasting learning. IPE is a combination of self and peer assessments. The use of multi-axial assessment, which combines self-assessment and peer assessment, must be addressed.

In Japan, each university seeks to provide education that makes the most of its uniqueness in training psychologists, who are required to collaborate with multiple professions. Research has shown that IPE can be effective even for a short period, provided that its methods and content are well-designed [28]. The results of this study showed that there was no improvement in readiness for IPE. In the future, there is room to devise IPE programs for the second year and beyond, taking advantage of the university’s unique features. It is important to develop an IPE program that can be effective even in a short period.

## Conclusions

Our longitudinal study revealed that, surprisingly, readiness for IPE remained stable even without further IPE exposure after the first year. This was likely due to the effective structure of our practical training courses and program content. Notably, interprofessional collaboration skills showed significant improvement by the fourth year of training. These findings suggest that IPE readiness can be sustained and interprofessional practice readiness can be developed even in the absence of continuous IPE, highlighting the potential for strategic IPE implementation and curriculum design to foster long-term learning outcomes. Additionally, our findings suggest that daily interactions with diverse professionals and students from the outset may be crucial for sustaining readiness for IPE. Furthermore, incorporating experiential learning and reflective practitioner models into non-IPE practicum settings may enhance interprofessional collaboration skills. However, given that IPE readiness did not show significant improvement in this study, we cannot conclusively demonstrate a sustained effect of our IPE intervention, highlighting the need for further research to optimize IPE strategies and outcomes.
